# “They say it’s fentanyl, but they honestly look like Perc 30s”: Initiation and use of counterfeit fentanyl pills

**DOI:** 10.1186/s12954-022-00634-4

**Published:** 2022-05-25

**Authors:** Raminta Daniulaityte, Kaylin Sweeney, Seol Ki, Bradley N. Doebbeling, Natasha Mendoza

**Affiliations:** 1 College of Health Solutions, Arizona State University, 425 N 5th Street Arizona Biomedical Collaborative, Room 121, Phoenix, AZ 85004 USA; 2 School of Social Work, Arizona State University, Phoenix, AZ USA; 3 Center for Applied Behavioral Health Policy, School of Social Work, Arizona State University, Phoenix, AZ USA

**Keywords:** Non-pharmaceutical fentanyl, Counterfeit pills, Heroin, Illicit pharmaceutical opioids, Drug initiation, Qualitative research, Arizona

## Abstract

**Background:**

Worsening of the overdose crisis in the USA has been linked to the continuing proliferation of non-pharmaceutical fentanyl (NPF). The recent wave of NPF spread in the USA has been fueled by an increased presence of counterfeit pills that contain NPF. This qualitative study aims to characterize the motivation and practices of counterfeit NPF pill initiation and use among individuals using illicit opioids in Arizona.

**Methods:**

Between October 2020 and May 2021, semi-structured interviews were conducted with 22 individuals meeting the following eligibility criteria: (1) 18 years or older; (2) residence in Arizona; and (3) use of illicit opioids in the past 30 days and/or opioid use disorder treatment in the past 12 months. Participants were recruited through referrals by a harm reduction organization, craigslist ads, and referrals by other participants. Interviews were conducted virtually via Zoom. Qualitative interviews were transcribed and analyzed thematically using NVivo.

**Results:**

Out of 22 participants, 64% were male, and 45% were ethnic minorities. Age ranged between 25 and 51 years old. Participants noted significant recent increases in the availability of counterfeit NPF pills (“blues,” “dirty oxys”) that were most commonly used by smoking. The majority indicated first trying NPF pills in the past year, and the first use often occurred in situations of reduced access to heroin or pharmaceutical opioids. Participant decisions to switch over to more frequent NPF pill use or to maintain some levels of heroin use were shaped by local drug availability trends and personal experiences with NPF effects. They were also influenced by conflicting views of social acceptability of pharmaceutical-like drugs, perceived harms of NPF in terms of overdose risks and increased difficulty of quitting, and perceived benefits of switching to the non-injection route of opioid administration (e.g., from injecting heroin to smoking NPF pills).

**Conclusion:**

Our findings highlight the need for the implementation of novel policy, treatment, and harm reduction approaches to address the growing unpredictability of drug supply and NPF pill-specific risks, attitudes, and behaviors.

## Introduction

Provisional data from the US Centers for Disease Control and Prevention (CDC) indicate that there were over 100,000 drug overdose deaths in the USA during the 12 months ending in April 2021, almost a 30% increase compared to the year before [1]. The worsening of the overdose crisis has been linked to the profound societal and economic impacts of the COVID-19 pandemic and continuing proliferation of non-pharmaceutical fentanyl, fentanyl analogs, and other novel synthetic opioids (NPF) [2–5]. The continuing spread of NPF-type drugs shows new geographic patterns with greater increases in the Western part of the country [6], including Arizona [7].

The new wave of NPF spread is linked to the increased presence of counterfeit pills [8, 9]. Counterfeit pharmaceuticals are fake pharmaceutical products that are manufactured illegally in clandestine laboratories using pill press machines that are easily accessible through online sources [10]. They are designed to look like legitimate pharmaceuticals but may contain fentanyl, other novel synthetic opioids, and/or other drugs [11]. First reports about the counterfeit pills containing fentanyl emerged in the initial phases of the NPF epidemic in 2014–2015 [12]. However, their presence remained relatively restricted. Instead, powder-type NPF, often sold as heroin or mixed with heroin, became increasingly available, gradually saturating illicit drug markets and in some regions nearly replacing heroin [2, 12–14]. Since 2020, many states across the USA have registered notable shifts in the illicit market dynamics as NPF is increasingly available in counterfeit pills, not just in powder form and/or as heroin [8, 9, 11].

Increases in counterfeit pill presence have been especially dramatic in Arizona. Located on important drug trafficking routes, Arizona is considered to be the “ground zero” of the evolving NPF epidemic [15]. According to the Arizona Criminal Justice Commission, between 2019 and 2020 retail-level seizures of counterfeit pharmaceutical pills that contain fentanyl increased 764% in Arizona [7]. Concurrently, NPF-positive overdose fatality cases in Arizona increased over 80% in 2020 compared to 2019 [16].

Despite the substantial uptick in the opioid overdose mortality in the Southwest, most prior studies on the NPF-related experiences among people who use illicit opioids (PWUO) were conducted in the eastern or midwestern states [13, 17–25]. Importantly, currently available information on the use of NPF in the form of counterfeit pills is largely limited to law enforcement statistics [8], clinical case studies, and media stories [12]. This qualitative study aims to detail and contextualize drug use practices, motivations, and experiences related to counterfeit NPF pill use among PWUO in Arizona.

## Methods

Individuals who use illicit opioids were recruited via social media posts, craigslist ads, referrals by a local harm reduction organization, and from other participants. To qualify, individuals had to meet the following criteria: (1) at least 18 years of age; (2) currently residing in Arizona; and (3) use of illicit opioids in the past 30 days and/or participation in substance use treatment for OUD in the past 12 months. Phone-based eligibility assessment was conducted first before scheduling an interview. Due to COVID-19 restrictions, all interviews were conducted using the Zoom platform. The study was approved by the Arizona State University Institutional Review Board.

A total of 36 individuals contacted the research team and participated in a phone-based eligibility assessment. Out of a total of 36 callers, 30 met eligibility requirements and were scheduled for a zoom-based meeting. Six individuals were not eligible because they did not use opioids (*n* = 2), were in long-term recovery from opioid use (*n* = 1), or did not reside in Arizona (*n* = 3). Out of a total number of 30 who were scheduled for an interview, seven did not keep their interview appointments. One respondent who passed phone-based eligibility and completed the interview was removed from the final sample because of inconsistencies that were identified in the interview responses. There was a final sample of 22 completed interviews between December 2020 and May 2021.

Interviews included both structured and semi-structured sections that were informed by prior research [24, 25]. The structured assessment collected sociodemographic and drug use history data. Semi-structured interview questions focused on the motivations and pathways of NPF and other opioid initiation, patterns of NPF, heroin and other drug use, perceived local availability of heroin, NPF and other drugs, perceived benefits and risks associated with NPF and other drug use, and COVID-19 impacts on drug use experiences and behaviors.

Participants were allowed to keep their cameras on or off, depending on their personal preference. All interview participants were compensated with a $40 e-gift card that was delivered via e-mail through the Tango rewards management system after completion of the interview session. Most interviews lasted about 50–60 min.

All audio-recorded interviews were transcribed and analyzed thematically using NVivo [26]. In the first stage of deductive coding, the team developed a coding schema based on the broad topics that were included in the interview guide (e.g., history of opioids use, NPF availability, first NPF use) [27]. In the next stage of inductive coding, the interview segments related to the history and patterns of NPF use were coded further by the first author to identify emerging codes and patterns. For example, second stage coding included the development of codes that capture reasons for the transition to NPF pill use (e.g., “easy availability of NPF”; “reduced access to heroin”; “reduced access to pain pills”; “switching to smoking”). The first and second authors met to review, compare, and discuss the coding. The coding process involved several rounds of labeling and re-organizing the data [27]. After the coding was completed, coded interview segments were queried using NVivo. Simultaneously, brief interview summaries and interpretive notes were completed in separate Excel sheets to compare key themes across all participants. This approach is based on the key principles of iterative categorization described elsewhere [28]. When writing the paper, NVivo-based queries that provide access to complete interview transcripts and summaries in Excel were used simultaneously to ensure a consistent and systematic approach to the interpretation and presentation of qualitative data [27]. All participant names used in this paper are pseudonyms.

## Results

### Participant characteristics

**Fig. 1:**
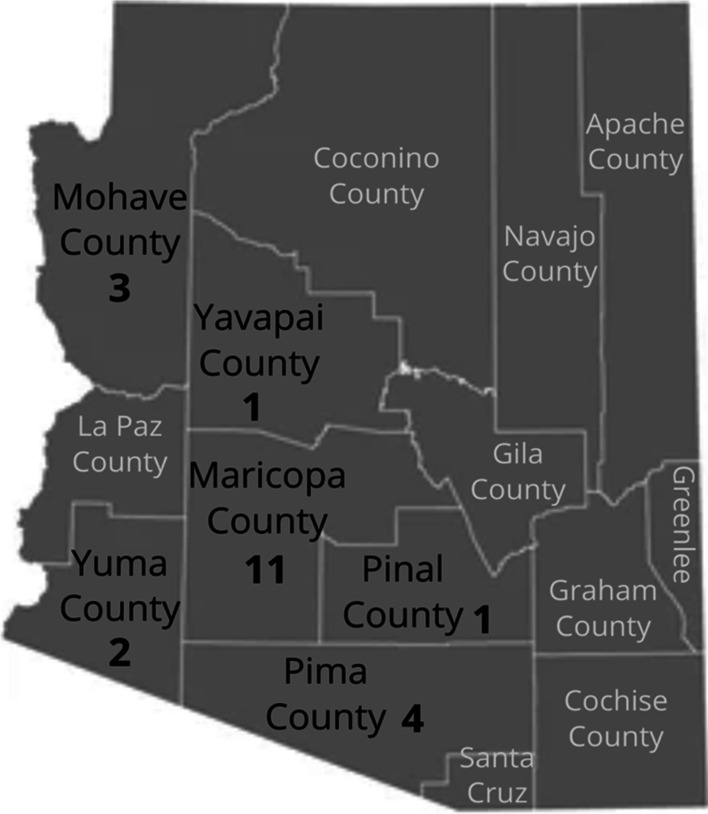
County of residence in Arizona of 22 participants recruited and interviewed for the study

Out of 22 participants, 14 were male (64%) and eight were female (36%). In terms of ethnic/racial background, 12 were non-Hispanic White, seven were of Hispanic/Latinx ethnic background, one was of Native American, one African-American, and one of mixed white and Asian ethnic background. Age ranged from 25 to 51 years old, with a mean of 33.9 years. The majority of participants (11, 50%) resided in Maricopa County (Phoenix area), four individuals were from Pima County (Tucson), and others were from Mohave, Yuma, Pinal, and Yavapai Counties (Fig. 1). Eighteen participants (82%) were unemployed and four reported current homelessness.

The majority reported use of both heroin and NPF pills in the past 30 days, with 82% reporting use of heroin on at least one day in the past 30 days and the same percentage (82%) reporting use of NPF pills. In terms of frequency of use, 14 individuals indicated that they used NPF more frequently than heroin, seven used heroin more frequently, and one person indicated only using non-prescribed pharmaceutical opioids. At the time of the interview, eight individuals were enrolled in opioid use disorder treatment (five in methadone, two in naltrexone, and one in buprenorphine-based treatment).

### Non-pharmaceutical fentanyl in Arizona

#### Non-pharmaceutical fentanyl in pressed pills

Nearly all participants described recent dramatic increases in the availability of non-pharmaceutical fentanyl (NPF) in the form of pressed pills mimicking 30 mg oxycodone, most commonly referred to as “blues,” “dirty oxys,” or fentanyl pills. Amy, a 31-year-old woman from Phoenix, who had a history of illicit pharmaceutical opioid use before transitioning to heroin at the age of 21 and then eventually to NPF pills at the age of 30, explained: “Well, they say it’s fentanyl, but they honestly look like Perc 30 s because…when I was doing Perc 30 s they looked exactly the same. And it says 30 on it.” Alex, a 43-year-old man who recently moved from Phoenix to his hometown in the northwestern part of Arizona to attend a methadone treatment center, also described the increased presence of NPF pills: “Okay, so, yeah, it was just an ingenious thing to make money that they did, making those counterfeit Perc 30 s. In Phoenix, they are absolutely everywhere.”

The increasing availability of NPF pills was associated with sharp declines in the prices of these pills. Ricky, a 42-year-old man from Phoenix, who started using NPF pills amid the COVID-19 pandemic and in the context of personal losses and turmoil in his life, explained the changes in prices between the summer 2020 and spring 2021:

There’s a mass amount [of “blues”]. The fact that when I first bought my first pills, it was $8. The way that it is now, it’s $5, shows you that the supply is flooding and outdoing the demand. They’re flooding the market so much that the pills are getting cheaper to buy.

Similarly, Kai, a 31-year-old man from the Tucson area (Pima County), explained his experiences: “Cause before, in the beginning, it would be like $10 for one pill, but now I would never wanna pay that much for one pill. It’s more like $5 a pill or $6.”

Increased street availability of NPF pills was often compared to the declining availability of heroin. Sofia, a 28-year-old woman from Tucson, noted, “It [heroin]’s been very difficult to find, and blues are everywhere. In the city I live in, they’re everywhere. That’s all anybody wants to do anymore is fentanyl.” Similarly, Jeff, a 40-year-old man from Phoenix who used illicit opioids to manage his pain issues, described his experiences: “Like my roommate, he's got to drive like eight miles to buy heroin when I could literally walk out in the apartment courtyard and find three or four people selling blues because the only reason I started doing it because it [blues] was cheaper and more accessible.”

Most participants noted that unlike the easy availability of counterfeit pills, access to genuine pharmaceutical opioids has declined sharply. Johnny, a 31-year-old man from Phoenix who used “blues” daily, characterized genuine pain pill availability: “The real ones [pain pills] are extremely, extremely hard to get. You have to get those prescribed, and I think only car accident and cancer patients can get prescribed real Percocet, you know what I mean, especially 30 mg.”

#### Non-pharmaceutical fentanyl in heroin

Several participants discussed the increasing presence of NPF in the local heroin supply. Powder-type heroin was viewed as being more prone to contamination with NPF. However, some individuals also noted the increasing presence of NPF in black tar heroin, which is the most common form of heroin available in the region. Lucas, a 26-year-old man from Mohave County described his experiences: “I mean, any time I get powdered heroin, then I know it’s fentanyl…. Even black tar heroin will have fentanyl in it, but it’s not as much. If it’s powdered heroin, then it’s pretty much just fentanyl.” Beth, a 40-year-old woman from Phoenix, also described prior experiences of obtaining heroin that was laced with fentanyl. She avoided it, though, by limiting her dealings to established and trusted sources:

I've had heroin like that [mixed with NPF]. You can usually taste it. A lot of dealers will let you know, “Hey, this has been laced with fentanyl because it gives you a better high…” I stay away from that. I have one particular dealer that I use that I have had for quite some time now. He's pretty honest with me with what he knows is going into it, and I stay away from it for the most part.

#### Non-pharmaceutical fentanyl in powder form

A few participants mentioned sporadic availability of powder-type NPF in Arizona, although it was viewed as far less common than NPF in counterfeit pill form. Jasmine, a 26-year-old woman from Phoenix, described her experiences of obtaining powder-form fentanyl, referred to as “pure”:

No, it's not [very available in Arizona]. It's really expensive. Most of my people that sell the pills don't also have access to the pure. But I've come across it quite several times and on it and then it is just kind of weird because some of the powder is white, some is pink, some is purple, and you're not really sure what you're getting. I guess, or why it's different colors. But it's great when you do find it. Some you can smoke it or shoot up and it's just really strong.

Antonio, a 25-year-old man from Yuma who had a prior history of selling NPF and other drugs, explained why local availability of NPF pills was greater than that of powder-form NPF, “Here […] for the most part the cartels are just pushing the pills. They don’t wanna sell the pure thing, or obviously, they’ll be killin’ the client base. For the most part, you only find pills out here…”.

### Initiation of counterfeit NPF pill use

Nearly all individuals reported the first use of pressed NPF pills in the past year, and all of them had prior experiences of using other illicit opioids. A few individuals noted that they first tried NPF pills because of the social circumstances that facilitate their access to the drug and sparked their curiosity about its effects. Maria, a 47-year-old woman from Mohave County, transitioned to heroin use three years ago after many years of using prescribed and illicit pain pills. She indicated that she first smoked NPF pills just a few months prior to the interview: “Unfortunately, I had a family member that was, um, is probably still currently involved in the trade of it, and like that through them. Socially, you know, ‘Here, you want to try it?’”.

More commonly, the first use of NPF pills occurred in the context of reduced access to heroin. In most situations, these initiation decisions were driven not by consumer preferences but by shifting market situations and circumstances. For example, Scott, a 30-year-old man, described his first use of NPF pills:

I live in Tucson now, but I actually ended up going back home—back to Scottsdale [city northeast of Phoenix] where I grew up—where my parents live. I went back for Christmas with my girlfriend at the time. We both did heroin. I got back to Scottsdale. We actually ran out of “black”—out of heroin—and we were trying to get more. I, actually, couldn’t find heroin. The people… that I’d known from growing up, didn’t have heroin. They had, actually, these pills. These fentanyl pills. They were actually the only thing I was able to get at that time. I ended up getting them. I wasn’t very happy about it. I didn’t really know about them. I knew about them, but in my opinion, I didn’t really care because I did heroin. I didn’t care to try anything else or try something different. Heroin worked for me. I didn’t really have a choice in the matter ‘cause I was gonna get sick. I actually ended up buying the fentanyl pills.

For a few individuals, the first use of NPF pills occurred through a pathway of reduced access to pharmaceutical opioids. Jeff, a 40-year-old man from Phoenix, explained how he was first introduced to NPF pills in the context of reduced access to legitimate pain pills that were prescribed to him after a serious work-related injury:

All the pain management places that you go to, now they're so afraid because of the opiate epidemic that they don't want to put you on what you need to be on… […] I think after being out of the nursing home for a few months, and they cut my pain pills down again, and [it] just wasn't enough. And I couldn't function, I couldn't walk when you have two shattered legs. And I think somebody ended up giving me one of those fentanyl pain pills, and you know it helped… it was way better than what I was getting from my pain management.

### Switching over to NPF pills or not (yet)?

Although nearly all individuals had tried NPF pills, participants noted distinct patterns and trajectories of switching over to NPF pills and/or maintaining some levels of heroin use. Some individuals transitioned to NPF use nearly immediately after trying them for the first time. For example, Scott explained, “Basically, from that moment on, is when I started seeking out the fentanyl pills and doing those on a daily. Then, basically, within a week I phased out heroin and was not shooting up anymore. I was just smoking these pills.”

In contrast, others made a slower transition by initially supplementing and then eventually replacing heroin with NPF pills over the course of several weeks or months. Johnny, a 31-year-old man, who was smoking about 10 NPF pills per day, explained: “Gradually, I started picking a couple of them up along with some heroin here at a time, and then after a while, I just started to like the pills better.” Others have maintained a varying pattern of dual-use, relying on NPF pills intermittently when heroin was more difficult to find, while still using heroin when available. Some individuals had tried using NPF pills but attempted to avoid them altogether and maintained a more or less consistent pattern of heroin use. For instance, Beth, a 40-year-old woman, who used heroin daily to help her with chronic pain, noted: “The pills I won't even touch honestly unless I haven't been able to find the heroin or I'm somehow in some type of withdrawal state is the only time that I'll actually smoke one of those pills. It's really just to get better until I can find the heroin.”

Participant decisions and processes of switching over to NPF pills or maintaining some levels of heroin use were shaped by a range of personal and contextual factors including availability trends, personal experiences with NPF effects, views about the social acceptability, as well as perceived benefits and risks of NPF versus heroin use.

#### Going with what is available

For most participants, their personal experiences with the availability and prices of NPF pills relative to heroin (or in a few cases to pharmaceutical opioids) were among the most important factors that determined their transition trajectories and patterns of NPF pill use. For example, Antonio noted that the COVID-19 pandemic “shorted the market on a lotta drugs and opened up the way for fentanyl.” As a result, these changes pushed many into NPF pill use:

I didn’t really start using [NPF pills] until last year [2020] and just because it was cheaper. I know a lotta people that never would’ve touched it [NPF pills], touched it last year. That’s just because we were locked in and couldn’t go anywhere. The drugs got frozen pretty much south of the border, and the only thing coming in were [NPF] pills.

Kai, a 31-year-old man from Pinal County who smoked NPF pills every day and heroin on about seven days in the past month, also noted that drug availability was one of the key factors in his transition to more frequent use of NPF pills: “Then the pills, they started just becoming more available. There was more of them than there was of the heroin, and they were cheaper.”

In contrast, Alex, a 43-year-old man, explained that he did try using NPF pills on several occasions, but maintained more frequent heroin use before starting methadone treatment. One of the underlying reasons for his continued more frequent use of heroin: “I actually met somebody that would sell it [heroin] to me fairly reasonable, and I could afford to be a heroin addict in Phoenix. I was doing it for $20 a gram there in Phoenix.” Similarly, Lucas, a 26-year-old man from Mohave County, had tried smoking NPF pills but considered heroin his drug of choice and injected it daily in the past 30 days. He reflected on the potential for him to eventually switch over to NPF pill use: “If I can’t find heroin, then I definitely will switch to blues, you know what I mean? I’ll probably end up doing that.”

#### Factoring in the effects of fentanyl

For many individuals, subjective experiences of fentanyl high were often discussed as another key factor that influenced their decisions and preferences concerning more or less frequent NPF pill use. Scott, a 31-year-old man, explained that in addition to the shifting availability trends, greater potency of NPF pills was one of the key motivators for his rapid transition from heroin to NPF pills: “Me and my girlfriend at the time, we smoked them, and they worked amazing. They got me higher than heroin did.” Antonio, a 25-year-old man, shared similar experiences: “Fentanyl was just more powerful to me. It calmed me down faster, got me in a high zone a lot faster than heroin did.”

In contrast, Maria, a 47-year-old woman, smoked NPF pills on a few occasions but was not impressed with their euphoric properties. She preferred to inject heroin and continued to use it more frequently:

The fentanyl is to me, I really didn't get a whole lot out of it personally. Then again, I didn't do a lot of it… It was once or twice in a probably a 4-5-day span and then that was it… I'm not continuing to use it at all, because I really didn't get a lot out of it.

Some individuals disliked the effects of NPF pills even as they were gravitating toward more frequent use because of easier accessibility. In comparison with heroin, fentanyl was viewed as more overpowering and disruptive to their daily functioning and routines. Sofia, a 28-year-old woman, who preferred heroin and, to the extent possible, sought to use it more frequently than NPF pills, explained her hesitancy to make a complete switch from heroin to NPF pills:

Everyone is doing blues. It makes people nod out. It puts people asleep—look like they’re sleeping all the time… I don’t enjoy spending my entire day with my head sunk between my legs, nodded out. That’s what fentanyl does to everybody, at least that I see. I don’t enjoy that. I believe that heroin is better for me to do if I’m gonna do it because I don’t nod out on heroin.

Similarly, Francisco, a 51-year-old man, used NPF pills more frequently than heroin because of easier access. However, he found fentanyl effects less attractive in comparison with heroin because of the shorter duration of action and the need to engage in more frequent use:

Well, because you only gotta do one-shot [of heroin] in the morning and you’re good all day. Fentanyl is one of them things. It’s like coke, man […] when you do smoke a fentanyl pill and you’re good for a little while, and then you gotta smoke another one. You’re good for a little while, and then the times get shorter and shorter...

#### A sense of familiarity

Some individuals felt that NPF pill resemblance to well-known pharmaceutical drugs contributed to the increased social acceptability and a sense of familiarity which made the transition easier and more likely. Alex, a 43-year-old man, described his initial reactions and experiences with increased NPF pill availability:

Well, fentanyl was just a weird thing how that whole thing happened. I told you I was familiar with Perc 30. I took a lot of those pills from my pain management doctor. When the cartels decided they were gonna start making bootleg Perc 30s using fentanyl, I liked it initially.

Similarly, Antonio, a 31-year-old man, explained how he and his peers viewed the introduction of NPF pills to the illicit drug market:

Naturally, [since] the fentanyl is introduced as a Percocet, Percocet 30 milligram, of course people who already were keen with liking those kinda drugs took a keen interest to fake 30s… [They] were already comfortable with the idea of taking it ’cause it was familiar to them.

#### Switching from injection to smoking

NPF pills in the form of pressed 30 mg oxycodone tablets were generally administered by smoking. Jaime, a 31-year-old man, explained his first experiences with NPF pills: “I remember, thinking it was weird that you put this pill on some tin foil and you smoke…. I didn't really care for the smell. I was still kind of all about heroin, but it did [work]…”.

For individuals who had been using heroin by injection, switching to NPF pills also meant a transition from the injection route of opioid administration to smoking. As a result, some felt that their switch to NPF pills was “a saving grace” because smoking was viewed as a less stigmatized and overall safer and more convenient alternative to the injection route of opioid administration. Johnny described his transition to NPF pills:

It was like my saving grace from a worse evil I guess you could say. Surprisingly, just one day… I put the needle and the heroin down, and I just started smoking [NPF pills]. I guess I could say it’s a matter of opinion, but I say it’s the lesser of the two evils when it comes to how you’re doing it as I think smoking is obviously a lot better than injecting.

Lucas had been using black tar heroin more frequently than NPF pills, but considered that switching the route of administration was one of the attractive aspects regarding his potential future switch to NPF pills: “You know, actually, I’ve heard that a lot of people got off shootin’ heroin by doin’ blues. They stopped shootin’ heroin by doing that, so I don’t know. Maybe it’s something I’ll get into… It’d be nice to not have to shoot up heroin anymore…”.

Others have also felt that the smoking route of NPF pill administration could help moderate their overdose risks, although in some situations these risk perceptions were difficult to reconcile with the observed NPF-related overdose experiences in the community. Scott reflected, “From my heroin addiction, people didn’t overdose when they smoked it. The only way to overdose was to do a huge shot of heroin. […] But now I’ve seen people overdose from smoking this fentanyl… I don’t really know how to weigh the risk.”

#### Risks of overdose and difficult to quit

Several participants expressed their concerns about the increased overdose risks associated with NPF use, and these fears deterred some individuals from making a complete switch over to NPF pills. As Alex, who used heroin more frequently than NPF pills, explained:

Most of those pills are stronger than a regular pharmaceutical Perc 30, and I liked that. I tended to stay more towards heroin. I did smoke my share of those pills. A lotta people die from 'em. That’s why it scared me.

Others were also concerned that NPF was more difficult to quit because of greater potency and more severe withdrawal symptomatology. Tahlia, a 26-year-old woman from Phoenix, discussed her concerns:

It’s very addictive. It’s a lot stronger than heroin so it’s a lot harder to get off of than heroin and that’s another thing that I’ve noticed. I’ve had a lot of people tell me that it’s almost impossible to get off of. That’s what I heard.

## Discussion

This is one of the first studies to describe PWUO experiences with increased availability and use of counterfeit NPF pills in the USA. Similar to prior studies on the initiation of NPF in powder form and/or mixed with heroin [13, 29], counterfeit NPF pill use was primarily driven by shifting illicit drug market conditions, not by consumer demands. However, since pharmaceutical drugs are generally viewed as less risky and stigmatized than heroin [30], our qualitative findings indicate that “repackaging” NPF-type drugs into counterfeit pharmaceutical-like products may contribute to perceptions of familiarity and increased social acceptability.

Importantly, our participants emphasized that counterfeit NPF pills in Arizona were generally used by a smoking route of administration, and for many, changes in the route of administration (from injecting heroin to smoking NPF pills) were viewed as a positive aspect of NPF transitions. No prior studies on heroin to NPF transitions conducted in the eastern part of the country have identified changes in the route of opioid administration that were described by our study participants. A recent study conducted in San Francisco, California, [31] was the first to characterize a pattern of replacing black tar heroin injection with a smoking method of NPF use. Similar to our participants in Arizona, PWUO in San Francisco viewed this transition as beneficial in terms of reduced stigma and health risks. Uniquely, PWUO in Arizona noted most commonly smoking NPF in a counterfeit pill form, while PWUO in San Francisco used powder NPF for smoking [31]. Our findings emphasize the need for ongoing tracking of unique adaptations and patterns of use that local communities of PWUO develop in response to the local specificities and situations of drug market conditions.

Participant views and experiences with the transition to the non-injection route of drug administration have significant implications for the development of harm reduction services. Prior research has established that the injection route of drug use is linked to greater drug-related harms (e.g., the transmission of HIV, hepatitis B and C, skin abscesses, and other bacterial infections) than other routes of drug administration [32–35]. Most prior public health interventions had limited success in helping individuals who inject drugs to switch to non-injection routes of drug use [32]. It is important to emphasize that smoking presents its health-related risks, including exacerbation of respiratory problems and potential exposures to a range of toxins and pathogens [33]. To respond to the changing drug use patterns in the community, it is critical for the local harm reduction services to expand their reach to individuals who use non-injecting routes of drug administration and provide education about safer smoking practices along with sterile smoking paraphernalia [31].

We acknowledge that these findings are based on the data derived from a small qualitative sample. Although we were able to recruit participants from distinct regions of the state (Fig. 1), and different ethnic minority groups, more research is needed with larger samples of participants to identify regional differences and specific needs of ethnic minorities in Arizona. Our findings are limited to self-reported data. In future research, it is critical to include urine toxicology analyses to identify specific fentanyl analogs and other novel synthetic drug exposures [14, 24]. The interviews were conducted via Zoom, and this might have limited our access to individuals who have less confidence in using online resources. On the other hand, our experiences suggest that videoconferencing platforms provide a novel and efficient format to engage PWUO in research. With a few exceptions, most of our participants had current or prior histories of heroin use. More research is needed to assess the use of other types of counterfeit pills (e.g., counterfeit Xanax bars) and NPF pill use among individuals without prior history of heroin use, including school-aged youth, since there were increased NPF-related overdose fatality cases in this population in Arizona [36].

## Conclusions

Our findings indicate the rapid proliferation of NPF in Arizona, primarily in counterfeit pill form, and are consistent with the law enforcement statistics [15] and overdose mortality data [36]. As heroin becomes less available and access to prescribed pharmaceutical opioids more restricted, NPF domination in the local drug markets has significant implications for policy modifications to allow the expanded use of drug checking technologies to identify the presence of NPFs in street drugs [12]. Although there is growing use of fentanyl testing strips as a harm reduction tool to qualitatively identify fentanyl and some analogs to help individuals adopt safer drug use practices [37–39], there is a need for community-based drug checking services and use of more advanced technologies that can be reliable and specific in identifying emerging fentanyl analogs and other novel drugs [40, 41].

From a community and clinical practice standpoint, there is an urgent need to address the structural and attitudinal barriers for patient access to life-saving medications for opioid use disorder [42]. Our interviews suggest that participants may have even more pessimistic attitudes toward their prospects of quitting NPF. These findings are consistent with prior studies [25] and suggest a need to “re-calibrate” existing buprenorphine-based and other opioid use disorder treatment protocols to address the unique needs of patients using NPF-type drugs [43–45].

From a research standpoint, it is imperative to continue examining the confounding influence of the COVID-19 pandemic on individuals who are at risks resulting from NPF as the combined crisis could lead to increased health disparities over time [46] especially for ethnic minority populations [47] and those experiencing homelessness [48]. Lastly, research efforts that seek to develop and test new means of community-wide surveillance will become critical [49] for the development of a more timely and geographically targeted delivery of harm reduction services and interventions.

## Abbreviations

NPF: Non-pharmaceutical fentanyl
OUD: Opioid use disorder
PWUO: People who use illicit opioids
